# (*Z*)-2-(1,3-Thia­zolidin-2-yl­idene)cyan­amide

**DOI:** 10.1107/S160053681002917X

**Published:** 2010-07-31

**Authors:** Xiao-tao Chen, Liang-zhong Xu

**Affiliations:** a College of Chemistry and Molecular Engineering, Qingdao University of Science and Technology, Qingdao 266042, People’s Republic of China

## Abstract

In the title compound, C_4_H_5_N_3_S, the tdihydrothiazole ring is almost planar, the maximum and minimum deviations being 0.188 (2) Å and 0.042 (3) Å, respectively. The crystal structure involves intermolecular N—H⋯N hydrogen bonds.

## Related literature

The title compound was synthesized as an inter­mediate for the synthesis of nicotine insecticides. For their biological activity and synthetic information, see: Jeschke *et al.* (2002 ▶); Hense *et al.* (2002 ▶). For a related structure, see: Dupont *et al.* (1995 ▶). For typical triple-bond lengths, see: Allen *et al.* (1987 ▶)
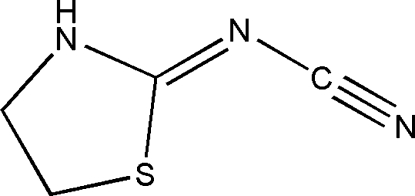


## Experimental

### 

#### Crystal data

C_4_H_5_N_3_S
*M* *_r_* = 127.18
Triclinic, 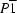

*a* = 6.4556 (13) Å
*b* = 6.5584 (13) Å
*c* = 6.7910 (14) Å
α = 83.28 (3)°
β = 81.53 (3)°
γ = 82.12 (3)°
*V* = 280.32 (10) Å^3^
*Z* = 2
Mo *K*α radiation
μ = 0.46 mm^−1^
*T* = 113 K
0.24 × 0.18 × 0.04 mm

#### Data collection

Rigaku Saturn diffractometer
Absorption correction: multi-scan (*CrystalClear*; Rigaku, 2005 ▶) *T*_min_ = 0.898, *T*_max_ = 0.982
1570 measured reflections
968 independent reflections
865 reflections with *I* > 2σ(*I*)
*R*_int_ = 0.039

#### Refinement

*R*[*F* ^2^ > 2σ(*F* ^2^)] = 0.039
*wR*(*F* ^2^) = 0.102
*S* = 1.06
968 reflections
73 parameters
H-atom parameters constrained
Δρ_max_ = 0.38 e Å^−3^
Δρ_min_ = −0.37 e Å^−3^

### 

Data collection: *CrystalClear* (Rigaku, 2005 ▶); cell refinement: *CrystalClear*; data reduction: *CrystalClear*; program(s) used to solve structure: *SHELXS97* (Sheldrick, 2008 ▶); program(s) used to refine structure: *SHELXL97* (Sheldrick, 2008 ▶); molecular graphics: *SHELXTL* (Sheldrick, 2008 ▶); software used to prepare material for publication: *SHELXTL*.

## Supplementary Material

Crystal structure: contains datablocks I, global. DOI: 10.1107/S160053681002917X/bv2145sup1.cif

Structure factors: contains datablocks I. DOI: 10.1107/S160053681002917X/bv2145Isup2.hkl

Additional supplementary materials:  crystallographic information; 3D view; checkCIF report

## Figures and Tables

**Table 1:** Hydrogen-bond geometry (Å, °)

*D*—H⋯*A*	*D*—H	H⋯*A*	*D*⋯*A*	*D*—H⋯*A*
N1—H1*C*⋯N3^i^	0.86	2.10	2.903 (3)	156

Symmetry code: (i) 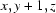.

**Fig. 1.**
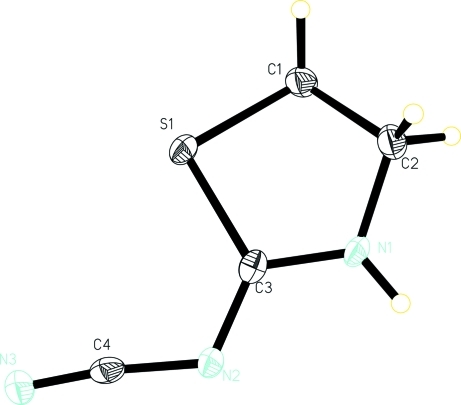
View of the title compound (I), with displacement ellipsoids drawn at the 40% probability level.

## supplementary crystallographic information

### Comment

Many nicotine insecticide derivatives have been reported showing various biological activities, *e.g.* thiacloprid (Jeschke *et al.*, 2002). The title compound (I) was synthesized as an intermediate for the synthesis of nicotine insecticides (Hense *et al.*, 2002). As an important intermediate for the syntesis of insecticides, we report here the crystal structure of (I).

In (I) (Fig. 1), main bond (C—C,*C*—N,*S*—C) lengths are normal and in a good agreement with those reported previously (Dupont *et al.*, 1995). Torsion angles of the thiazole ring are small [C4—N2—C3—S1, 6.52 (3)°, C1—S1—C3—N1, 9.95 (2)° and C2—N1—C3—S1, 7.98 (3)°] and thiazole ring is almost planar as the maximum and minimum deviations are 0.188Å and 0.042Å respectively. In the thiazole ring, the dihedral angle between plane A (C1/C2/C3/C4) and plane B (S1/N1/N2/C3) is 5.5 (3)°. The molecule contains a nitrile group, with an C≡N distance of 1.158 (1) Å, which indicates substantial triple bond character (Allen *et al.*, 1987). Recently, compounds containing the thiazolidin-2-yl-cyanamide group have attracted much interest because compounds containing a thiazole ring system are well known as efficient insecticides (Hense, *et al.*, 2002). The structure is stabilized by hydrogen bonds of N—H···N type.

### Experimental

Cyano-dimethyl dithiocarbamate 14.6 g (0.1 mol) was dissolved in 35 ml ethanol with stirrer and 2-amino-ethanethiol 11.4 g (0.1 mol) was slowly added to the mixture while maintaining the temperature at 303–313 K. After three hours, ethanol was removed under reduced pressure to give title compounds 11.2 g, yield 88%. (Jeschke, *et al.*,2002). Single crystals suitable for X-ray measurement were obtained by recrystallization from the mixture of acetone and methanol at room temperature.

### Refinement

All H atoms were placed in calculated positions, with C—H = 0.97 Å and N—H = 0.86 Å, and included in the final cycles of refinement using a riding model, with *U*_iso_(H) = 1.2*U*_eq_(C, N).

### Crystal data

**Table:**

C_4_H_5_N_3_S	*Z* = 2
*M**_r_* = 127.18	*F*(000) = 130
Triclinic, *P*1	*D*_x_ = 1.495 Mg m^−^^3^
Hall symbol: -P 1	Mo *K*α radiation, λ = 0.71073 Å
*a* = 6.4556 (13) Å	Cell parameters from 916 reflections
*b* = 6.5584 (13) Å	θ = 3.1–27.4°
*c* = 6.7910 (14) Å	µ = 0.46 mm^−^^1^
α = 83.28 (3)°	*T* = 113 K
β = 81.53 (3)°	Prism, colorless
γ = 82.12 (3)°	0.24 × 0.18 × 0.04 mm
*V* = 280.32 (10) Å^3^	

### Data collection

**Table:**

Rigaku Saturn diffractometer	968 independent reflections
Radiation source: rotating anode	865 reflections with *I* > 2σ(*I*)
confocal	*R*_int_ = 0.039
ω scans	θ_max_ = 25.0°, θ_min_ = 3.1°
Absorption correction: multi-scan (*CrystalClear*; Rigaku, 2005)	*h* = −6→7
*T*_min_ = 0.898, *T*_max_ = 0.982	*k* = −7→6
1570 measured reflections	*l* = −8→7

### Refinement

**Table:**

Refinement on *F*^2^	Primary atom site location: structure-invariant direct methods
Least-squares matrix: full	Secondary atom site location: difference Fourier map
*R*[*F*^2^ > 2σ(*F*^2^)] = 0.039	Hydrogen site location: inferred from neighbouring sites
*wR*(*F*^2^) = 0.102	H-atom parameters constrained
*S* = 1.06	*w* = 1/[σ^2^(*F*_o_^2^) + (0.0574*P*)^2^] where *P* = (*F*_o_^2^ + 2*F*_c_^2^)/3
968 reflections	(Δ/σ)_max_ < 0.001
73 parameters	Δρ_max_ = 0.38 e Å^−^^3^
0 restraints	Δρ_min_ = −0.36 e Å^−^^3^

### Special details

**Table:**

Geometry. All e.s.d.'s (except the e.s.d. in the dihedral angle between two l.s. planes) are estimated using the full covariance matrix. The cell e.s.d.'s are taken into account individually in the estimation of e.s.d.'s in distances, angles and torsion angles; correlations between e.s.d.'s in cell parameters are only used when they are defined by crystal symmetry. An approximate (isotropic) treatment of cell e.s.d.'s is used for estimating e.s.d.'s involving l.s. planes.
Refinement. Refinement of *F*^2^ against ALL reflections. The weighted *R*-factor *wR* and goodness of fit *S* are based on *F*^2^, conventional *R*-factors *R* are based on *F*, with *F* set to zero for negative *F*^2^. The threshold expression of *F*^2^ > σ(*F*^2^) is used only for calculating *R*-factors(gt) *etc*. and is not relevant to the choice of reflections for refinement. *R*-factors based on *F*^2^ are statistically about twice as large as those based on *F*, and *R*- factors based on ALL data will be even larger.

### Fractional atomic coordinates and isotropic or equivalent isotropic displacement parameters (Å^2^)

**Table:**

	*x*	*y*	*z*	*U*_iso_*/*U*_eq_	
S1	0.19920 (8)	0.40549 (8)	0.26592 (8)	0.0167 (3)	
N1	0.4033 (3)	0.7184 (3)	0.2385 (3)	0.0161 (4)	
H1C	0.5069	0.7914	0.2164	0.019*	
N2	0.6225 (3)	0.4094 (3)	0.2613 (3)	0.0158 (5)	
N3	0.6775 (3)	0.0290 (3)	0.2589 (3)	0.0234 (5)	
C1	0.0574 (4)	0.6574 (3)	0.2008 (4)	0.0184 (5)	
H1A	0.0483	0.6789	0.0584	0.022*	
H1B	−0.0843	0.6700	0.2732	0.022*	
C2	0.1853 (4)	0.8129 (3)	0.2610 (4)	0.0187 (5)	
H2A	0.1700	0.9417	0.1752	0.022*	
H2B	0.1384	0.8415	0.3985	0.022*	
C3	0.4340 (4)	0.5138 (3)	0.2539 (3)	0.0139 (5)	
C4	0.6407 (3)	0.2063 (3)	0.2601 (3)	0.0166 (5)	

### Atomic displacement parameters (Å^2^)

**Table:**

	*U*^11^	*U*^22^	*U*^33^	*U*^12^	*U*^13^	*U*^23^
S1	0.0153 (4)	0.0143 (4)	0.0224 (4)	−0.0053 (2)	−0.0049 (2)	−0.0020 (2)
N1	0.0159 (10)	0.0126 (10)	0.0221 (11)	−0.0057 (7)	−0.0056 (8)	−0.0021 (7)
N2	0.0160 (10)	0.0118 (10)	0.0211 (11)	−0.0035 (7)	−0.0050 (8)	−0.0022 (7)
N3	0.0211 (11)	0.0139 (11)	0.0358 (13)	−0.0013 (8)	−0.0083 (9)	−0.0013 (8)
C1	0.0154 (11)	0.0183 (12)	0.0227 (13)	−0.0004 (9)	−0.0065 (9)	−0.0034 (9)
C2	0.0200 (12)	0.0145 (12)	0.0221 (13)	−0.0003 (9)	−0.0050 (9)	−0.0039 (9)
C3	0.0192 (11)	0.0144 (11)	0.0096 (11)	−0.0054 (9)	−0.0031 (9)	−0.0018 (8)
C4	0.0126 (11)	0.0220 (13)	0.0166 (12)	−0.0041 (9)	−0.0053 (9)	−0.0008 (9)

### Geometric parameters (Å, °)

**Table:**

S1—C3	1.747 (2)	N3—C4	1.155 (3)
S1—C1	1.816 (2)	C1—C2	1.522 (3)
N1—C3	1.323 (3)	C1—H1A	0.9700
N1—C2	1.452 (3)	C1—H1B	0.9700
N1—H1C	0.8600	C2—H2A	0.9700
N2—C3	1.317 (3)	C2—H2B	0.9700
N2—C4	1.321 (3)		
			
C3—S1—C1	91.15 (10)	N1—C2—C1	106.10 (17)
C3—N1—C2	116.32 (19)	N1—C2—H2A	110.5
C3—N1—H1C	121.8	C1—C2—H2A	110.5
C2—N1—H1C	121.8	N1—C2—H2B	110.5
C3—N2—C4	117.9 (2)	C1—C2—H2B	110.5
C2—C1—S1	105.21 (16)	H2A—C2—H2B	108.7
C2—C1—H1A	110.7	N2—C3—N1	122.1 (2)
S1—C1—H1A	110.7	N2—C3—S1	125.53 (17)
C2—C1—H1B	110.7	N1—C3—S1	112.34 (17)
S1—C1—H1B	110.7	N3—C4—N2	173.3 (2)
H1A—C1—H1B	108.8		
			
C3—S1—C1—C2	−23.42 (16)	C2—N1—C3—N2	−171.4 (2)
C3—N1—C2—C1	−25.8 (3)	C2—N1—C3—S1	7.9 (2)
S1—C1—C2—N1	30.4 (2)	C1—S1—C3—N2	−170.6 (2)
C4—N2—C3—N1	−174.6 (2)	C1—S1—C3—N1	10.19 (17)
C4—N2—C3—S1	6.3 (3)	C3—N2—C4—N3	177 (2)

### Hydrogen-bond geometry (Å, °)

**Table:**

*D*—H···*A*	*D*—H	H···*A*	*D*···*A*	*D*—H···*A*
N1—H1C···N3^i^	0.86	2.10	2.903 (3)	156

Symmetry codes: (i) *x*, *y*+1, *z*.
